# Assessing the Aftermath of COVID-19 Outbreak in the Agro-Food System: An Exploratory Study of Experts' Perspectives

**DOI:** 10.3389/fnut.2022.769626

**Published:** 2022-04-15

**Authors:** Elena Raptou, Konstadinos Mattas, Efthimia Tsakiridou, George Baourakis

**Affiliations:** ^1^Department of Agricultural Development, Democritus University of Thrace, Orestiada, Greece; ^2^Department of Agricultural Economics, Aristotle University of Thessaloniki, Thessaloniki, Greece; ^3^Mediterranean Agronomic Institute of Chania, Chania, Greece

**Keywords:** COVID-19, factor analysis, food expert clusters, agro-food production, food supply networks, food consumption emerging trends

## Abstract

The present study explored COVID-19 outbreak impacts on the food system in terms of agro-food production, distribution networks efficiency, and emerging food consumption patterns according to food experts' perspectives. Individual level data were selected from a sample of 59 executive managers of different domains representing agro-food businesses, agro-food cooperatives, and agro-food consulting firms and public institutions. The empirical analysis addressed the effects of the COVID-19 crisis to all the stages in the food chain and attempted to indicate the factors that could influence the trajectory from “farm to fork” under uncertain circumstances. Factor analysis elicited the underlying dimensions of experts' viewpoints toward the operation of the food system during COVID-19 pandemic. Data were also elaborated through hierarchical and k-means cluster analysis and the cluster structure was further validated by discriminant analysis. A two-cluster solution emerged, revealing differences in experts' perceptions toward the aftermath of the pandemic on agriculture (socioeconomic impacts on rural areas, impacts on agricultural production), food processing businesses (decline in the economic viability of food businesses, sharp economic downturn in the food industry, economic recession, incentives for innovation), food distribution networks (distribution channels fallout, food supply disruption), and consumers' food habits and preferences (increasing interest in health protection, adoption of unhealthy eating habits, demand for innovative and sustainable foods). These segments were identified as “skeptical food experts about COVID-19 impacts” (33.9%) and “alarmed food experts about COVID-19 impacts” (66.1%). Our findings highlighted the main disruptions that the food sector should overcome to meet consumer demand for safe and healthy food products and also ensure food availability and food system resiliency.

## Introduction

The novel coronavirus disease, widely known as COVID-19 and caused by the SARS-CoV-2 beta coronavirus virus, started as a localized zoonotic outbreak in China in December 2019 and was announced as a pandemic by the World Health Organization in March 2020 (1). In order to support the health care systems and “flatten the curve,” governments enforced strict measures to ensure social distancing, including mandatory quarantine, border shutdowns and travel restrictions, large-scale electronic surveillance and closure of schools, workplaces, and transit systems (2). Declared as a “black swan” event (3, 4), the COVID-19 pandemic has spread quickly across 185 countries in six continents resulting in an unprecedented health and socioeconomic crisis. As of 18 April 2021, there have been 140,322,903 globally confirmed cases of COVID-19 and 3,003,794 deaths reported to World Health organization (5). In addition, the strict lockdown measures imposed to offset the health impacts have caused tremendous disparities in all aspects of the economy (6–8).

The new pandemic has created instability in the food sector, which has to confront new challenges from supply chain disruptions and their effects on the food system (9) to health protection of the workforce and maintenance of food availability, accessibility and usage (8, 10). COVID-19 pandemic threatened the production systems and governments were enforced to critically evaluate and regulate agro-food policies to ensure food supply availability and affordability to the public (11, 12). Recent literature underlined the special nature of the food industry and noted that food organizations differ from other organizations because of their “daily life-essential” products, making it imperative to avoid disruptions in the food chain (13, 14).

The food sector in Greece has a vital role since it accounts for one-third of total revenue of the manufacturing sector and its workforce represents 36% of total employment (15). During the last decade, the food processing industry has experienced the impacts of a severe economic recession and had to make adjustments for adapting to the new economic reality (16). Today, the food sector has to overcome the pandemic obstacles while upholding the safety standards, dealing with the constant lockdowns in food business and maintaining sustainability practices to the fore (17). Food businesses have to respond to consumers' awareness for increasing food safety, their turn to online delivery food services and their preference for long-shelf-life foods (18). Since consumers' time for in-store food shopping is limited, food stockpiling behaviors have arisen to mitigate consumers' fear for potential food shortage (19).

The present study sought to investigate the COVID-19 pandemic crisis impacts on the food industry in terms of agro-food production, distribution networks efficiency and consumers' purchase behavior and attitudes toward the new turbulent food environment. To shed light into the pandemic aftermath on food sector, food experts were asked to express their perceptions toward COVID-19 consequences on agricultural and food production, rural welfare, agro-food business operation, food distribution and delivery networks, and emerging individuals' food preferences and consumption patterns. Individual level data were selected from a sample of experts that were the executive managers of Greek agro-food businesses. The empirical analysis addressed the effects of the COVID-19 crisis to all the stages in the food chain and attempted to indicate the factors that could influence the trajectory from “farm to fork” under uncertain circumstances. Although vaccination to protect against COVID-19 virus is in progress, it is impossible to ignore potential repeated population infections and lockdown waves in the near future due to the ongoing pandemic breakouts till the end of 2021, putting further pressure on the food industry (20, 21).

## COVID-19 Pandemic Crisis and the Food Industry

To diminish COVID-19 infections within the food supply environment, appropriate response plans were designed to secure and direct the operations of the food supply chain at the time of the outbreak. Response plans included various control requirements for cleaning, sanitization, disinfection of facilities, and monitoring and screening of the food workers and supervisors to prevent COVID-19 transmission (22). It was of major importance for food businesses to protect employees' health during this pandemic crisis in order to retain the sufficient workforce for their operation and manage employees' unplanned absenteeism due to COVID-19 infections (13, 23).

Since food security depends on both agro-food production and trade, a robust supply chain is necessary to transport and deliver food products where consumers are. During the last year, COVID-19 pandemic restrictions have disordered the agro-food system by disrupting both production and distribution. The workforce health preventive measures have resulted in labor shortages in the food industry, especially within downstream food processing and distribution systems due to worker illness, self-isolation, or travel restrictions (24, 25). Furthermore, the majority of food and beverage processing business are small and medium-sized operations in terms of their staff size and constitute over 95% of firms in the food sector (26, 27). To the extent that small and medium sized-enterprises are more labor intensive than large-sized enterprises, they may be more vulnerable to labor disruptions (24). Small and medium sized enterprises are usually deficient in financial or managerial resources and experience difficulties in adjusting to disruptions, especially in case they keep on longer than expected (28–30). The fact that the small and medium-sized food businesses usually make decisions based on routine transactions and depend on a small number of customers increase the risks for raw material and stock shortages, production slowdown, and economic shrinkage (30, 31). The reduced productivity, or even closures, in food processing and distribution plants have in turn resulted in backlogs in farms with negative consequences for the harvest management, agricultural production, and animal welfare (24).

Therefore, the inconsistency in the food system due to problems in food production, distribution and delivery during the pandemic has eventually impacted the availability and access of food products to consumers (9, 12). Further obstacles in the availability of agro-food products were imposed from the closure of hotels and restaurants resulting in considerable reductions in food donations to food banks. This put additional pressure to the food industry, which had to meet the increasing demand as people suffered loss of incomes (24). In addition, the pandemic crisis has shaken consumers' confidence in the resilience of the food system. It remains a vital question for the food industry how consumers' purchase behavior has been affected after having seen empty store shelves in food stores at the initial stages of the pandemic (25). Food hoarding was a common practice during the lockdowns since consumers felt insecure about food availability and subsequent food price hikes. Reasons of food hoarding might stem from both rational and irrational motives. From the rational side, consumers might wish to stockpile essential food products for the 2-week-home-quarantine period in case of infection or tried to avoid transportation to food stores. To minimize personal contacts and maintain social distancing, consumers also increased purchases of food items with long shelf-life or takeaway meals (32). From the irrational side, peer influence played the key role since consumers seemed to imitate and follow others' consumption patterns, the so called herd effect (33). The irrational hoarding was the result of panic buying behavior and further complicated food shortages by putting extra burden in the food chain (4). Feelings of instability, uncertainty, and precariousness also incited panic purchase behaviors in an attempt to decrease anxiety and fear (34–36).

## Methodology

### Data and Sample Selection

To shed light into experts' perceptions toward the impact of COVID-19 pandemic on the food industry, this study employed a hybrid approach and incorporated both qualitative and quantitative research designs. The novelty of the specific topic of interest complicated the proper definition of the important elements for establishing the survey variables (37). Therefore, a qualitative approach could offer a deep understanding of experts' views on the impact of COVID-19 on food industry. The descriptive nature of the qualitative research could provide a primary insight into the research objectives through the unique viewpoints of those who have directly experienced the impact of the pandemic on the food chain (38, 39). Owing to the paucity of literature in this specific area, we also sought to build a holistic picture of COVID-19 economic and infrastructure impacts on the food industry by establishing a consensus of experience from food experts and stakeholders. Therefore, the qualitative research design included semi-structured interviews (40) that were held with eight food industry experts in May 2020 in order to provide a better understanding of COVID-19 consequences on food systems. An informal questionnaire with 15 open-ended questions was developed for the qualitative research interviews, whereas the sample selection was based on purposive sampling and snowballing techniques (41). The average interview duration was 50 min ranging from 34 to 93 min.

The findings of the qualitative research were used for the construction of the formal online questionnaire employed in the subsequent quantitative research design. The quantitative research involved three district stages, namely questionnaire design, sampling procedure, and data elaboration. The questionnaire design was mainly of a closed response format and considered experts' perceptions on the impact of COVID-19 crisis on agricultural and food production, food supply and distribution channels, consumer preferences, and respondent's agro-food business/organization operation and activity. It also included scale-questions for rating experts' views on food industry's initiatives for public health protection and contextual information on respondent's area of expertise and business personnel. The average time for the questionnaire completion was 15 min. The final questionnaire was pre-tested on a limited sample of food experts, who agreed to complete it and comment on its comprehensibility and clarity of questions, technical performance, and usefulness of instructions (42).

In the context of the present study, food experts were defined as stakeholders or agents with professional interest on varied backgrounds in the agro-food sector. To identify the sample, a generic list was adopted (43, 44) in order to consider potential survey participants. The key element was to obtain a target population that could represent the food industry and would minimize biased conclusions. We invited to participate in this survey a total of 102 executive managers from 102 different domains (i.e., different businesses, organizations, etc.), representing the following groups: (a) agro-food businesses (i.e., farm producers and food processing businesses), (b) agro-food cooperatives, and (c) agro-food consulting firms and public institutions. All business/organizations had been established for more than 15 years, whereas the great majority was small-sized and medium-sized enterprises of up to 249 employees (91.4%). Furthermore, all participants held an MSc certification and had a professional experience of at least 10 years in the agro-food sector (45). Recruitment was purposive and participants engaged in the survey voluntarily, with no specific reward after personal invitation. All the list members were invited to participate via email and were informed on the main purposes and stages of the survey (46, 47). The link to access the online survey and response confidentiality were provided within the email context. To achieve the maximum response rate, two reminding emails were sent with a 10-days frequency in those cases where no reply had been received, insisting on the importance of participation. Finally 59 completed questionnaires were selected between June and July 2020, corresponding to a response rate of ~58% for this online survey. The response rate is quite satisfactory and significantly higher to the average response rate of 11% for the online surveys (48).

### Measures

The formal questionnaire comprised the main data selection tool and was administered online to a specific sample of executive managers in the agro-food sector. Due to the dearth of the relevant literature and the novelty of the study, the questionnaire development and the variable construction were based on the outcomes of the precedent qualitative research (49, 50).

In the first part of the questionnaire, participants were asked to assess the COVID-19 consequences on the activities and practices of the agro-food businesses/organizations. In particular, participants had to state whether COVID-19 pandemic had affected the activity of the business/organization in which they were employed. A dichotomous indicator was constructed taking the value 1 in case of a positive response and zero otherwise. Second, a five-point scale ranging from “not at all” to “extremely” (not at all-slightly-moderately-very much-extremely) was used to express the extent to which participants believe that the operation of the agro-food business/organization will be affected in the future. Then, respondents had to specify the aftermath of COVID-19 pandemic by responding to an ordered variable taking the value 1 if the economic performance of the business/organization was expected to deteriorate, the value 2 for a neutral impact (no effect), and the value 3 in case the economic performance improved in the near future.

In the second part of the questionnaire, multi-item scales were employed to describe experts' perceptions toward the impact of COVID-19 on the food sector. To explore experts' viewpoints on the consequences of the pandemic on agricultural produce, rural areas welfare, and employment in the agricultural sector, participants were asked to score an 11-item Likert type scale rating from “totally disagree” to “totally agree” (totally disagree-disagree-neither disagree nor agree-agree-totally agree). This scale is presented in **Table 2**. Furthermore, a 14-item variable rated on a five-point Likert scale (totally disagree-disagree-neither disagree nor agree-agree-totally agree) was employed to investigate experts' views on the impact of COVID-19 on the food manufacturing industry, including food production, food processing businesses efficacy, and food innovation (**Table 4**). The effects of the pandemic on food supply and the efficiency of the distribution networks were expressed through a 9-item 5-point Likert type scale ranging from “totally disagree” to “totally agree” (**Table 6**).

The third part of the questionnaire focused on consumer food habits and preferences and how they were influenced by COVID-19 crisis. To assess experts' opinions on the impact of the pandemic on consumer demand, a 13-item 5-point scale was adopted ranging from “extremely unlikely” to “totally likely” (extremely unlikely-unlikely-neutral-likely-extremely likely”) (**Table 8**).

Finally, contextual information was included in the last part of the questionnaire. Respondents were asked to declare their employment in the agro-food business/organization on a categorical variable taking the value of 1 for “farmers,” the value of 2 for “food processing enterprises,” the value of 3 for “food distributors and sellers,” and the value of 4 for “consultants and policy makers.” The personnel size was expressed through an ordered indicator taking the value 1 for micro enterprises (up to 10 employees), the value 2 for small-sized enterprises (11–49 employees), the value 3 for medium-sized enterprises (50–250 employees), and the value 4 for large enterprises (over 250 employees).

### Methods of Analysis

#### Factor Analysis

Exploratory factor analysis (EFA) is widely used in social sciences to (i) assess theories of learning, cognition, and personality, (ii) explore scale validity, and (iii) decrease dimensionality in a set of variables so that they can be more easily adapted in further statistical analysis (51–53). In the present study, EFA was employed to test the potential interdependencies among the observed variables and the underlying theoretical constructs referred as latent variables (54). In particular, EFA was used to uncover the underlying structure of the multi-item variables expressing experts' opinions on the impact of COVID-19 crisis on agro-food sector and reduce it into smaller sets to test construct validity and enhance the interpretability of the multi-item scales (55–57).

Prior to EFA implementation, several prerequisites were worked out before proceeding to the analysis. First, suitability of the data was highlighted since the sample size might be considered as arguably small (*N* = 59). Recent literature has suggested that in case over-determination is strong and communalities are high (over 0.60), even relatively small sample sizes can assure accurate EFA solutions (58–61). According to Sapnas and Zeller (62), even sample sizes of 50 individuals can be appropriate for performing EFA. Furthermore, MacCallum et al. (63, 64) noted that all items in a factor model should have communalities of over 0.60 or an average communality of 0.7 to justify EFA application to small sample sizes. To evaluate the quality of the present study, the average communality was assumed to be at least 0.7. In addition, the Kaiser–Meyer–Olkin (KMO) criterion of sampling adequacy and the Bartlett's test of sphericity were adopted to assess the suitability of sample sizes for factor analysis and the fitness of the data (60, 65). KMO estimations over the value of 0.50 depicted data adequacy (66) and the Bartlett's test of sphericity was statistically significant for ensuring the suitability of EFA (60). Extracting factors with eigenvalues over 1 were retained according to K1 rule, whereas extracting factors with eigenvalues <1 were discarded without losing much of the original variability (67). To achieve the optimal factor solution, all variables with estimated factor loadings over 0.40 on more than one factor were removed from the analysis (68). Finally, reliability analysis (Cronbach's α coefficient >0.60) was conducted to measure the unidimensionality of the set of the variables representing each factor (69).

For the present study, five EFA applications were considered to determine the underlying components that explain experts' perceptions toward the impact of COVID-19 pandemic on agricultural production, food processing businesses, food supply and distribution networks, purchase behavior, and food industry initiatives for public health safety. Principal component analysis (PCA) was employed with the Varimax rotation method for aggregating variables that load highly on a specific factor.

#### Cluster Analysis

In order to group experts according to their perceptions toward COVID-19 pandemic and its impacts on the food industry, cluster analysis was conducted for providing similar expert segments based on internal homogeneity and intragroup heterogeneity. Each cluster was mutually exclusive with the maximum differentiation between clusters and the maximum homogeneity within each cluster (70). Since this study focused on participants' classification rather than building a predictive model, cluster analysis was suggested as the most appropriate tool to assign experts into different homogenous segments and ascertain differences in perceptions toward the aftermath of COVID-19 in the agro-food sector (71–73).

The clusters were defined by both hierarchical and non-hierarchical (k-means) clustering techniques. First, hierarchical cluster analysis was employed to define the adequate number of clusters after classifying cases into homogenous clusters by combining them together one at a time in a series of sequential steps (74, 75). The Ward's method was used as the agglomeration method to determine the optimal number of clusters. In Ward's method, cases are combined so as to ensure the lowest increase of the variance in the cluster, and hence its highest homogeneity (76). The squared Euclidean distance was adopted as a measure of similarity between cases. Second, the validity of hierarchical clustering was enhanced by k-means algorithm, in which it was set a priori the number of clusters resulted from the Ward's hierarchical clustering method. Finally, the cluster structure was further validated by discriminant analysis, which estimated a discriminant function for examining the accuracy by which the study participants were allocated in the clusters (77, 78).

## Results

Table 1 depicts the profile of food industry experts engaged in the present study. Of the 59 participants, 27.1% were farmers (and also the managers of the farms), 33.9% were executive managers in food processing enterprises, 25.4% were food distributors and sellers, and the rest 13.6% corresponded to consultants and policy makers. In total, the great majority of experts (43.1%) were engaged in micro enterprises of up to 10 employees, whereas the 86.1% were the managers of small and medium-sized enterprises (SME). According to recent evidence, SMEs constitute the overwhelming majority of Greek enterprises, corresponding to 85% of private employment and have suffered the most by the prolonged economic recession in the last decade (79). Approximately 93% of respondents stated that COVID-19 pandemic has already affected the economic activity of the enterprise in which they are employed with almost 78% predicting that the economic performance of food businesses will deteriorate in the near future.

**Table 1:** Experts' profile (*N* = 59).

**Variable**	**Frequency**
**Occupation**	
Farmers	27.1%
Food processing enterprises	33.9%
Food distributors—sellers	25.4%
Consultants—policy makers	13.6%
COVID-19 pandemic has affected the business/organization I work for	93.2%
**To what extent do you think the activity of the business/organization in which you are employed will be affected by COVID-19 pandemic?**	
Not at all	6.8%
Slightly	5.1%
Moderately	28.8%
Very much	44.1%
Extremely	15.5%
**How the economic performance of the business/organization, in which you work, will be affected in the near future?**	
It will deteriorate	78.0%
It will not be affected	10.2%
It will improve	11.9%
**Personnel (number of employees)**	
Micro enterprises (up to 10 employees)	43.1%
Small-sized enterprises (11–49 employees)	27.6%
Medium sized enterprises (50–250 employees)	17.2%
Large enterprises (over 250 employees)	12.1%

### The Impact of COVID-19 Crisis on Agricultural Production

Participants' viewpoints on the consequences of COVID-19 on agricultural production and Greek farms are described on Table 2. Agro-food experts seemed to agree that COVID-19 crisis will affect agricultural production (44.06%) and may exacerbate inequalities between farmers (52.54%), although the productivity of Greek farms is not expected to be influenced to a greater extent compared to other countries (47.46%). The stability of agricultural incomes was also questioned since a great proportion of respondents stated that agricultural income decline is inevitable (45.76%) and rural areas will experience significant job losses as an aftereffect of the pandemic (32.2%).

**Table 2:** Experts' perceptions toward COVID-19 impacts on agriculture (%).

**Variables**	**Strongly disagree**	**Disagree**	**Neither disagree nor agree**	**Agree**	**Strongly agree**
COVID-19 pandemic will affect agricultural production	5.08	10.17	40.68	28.81	15.25
COVID-19 pandemic will cause significant shortages of raw materials used in agricultural production	8.47	33.90	30.51	15.25	11.86
COVID-19 crisis will cause a considerable reduction in agricultural production	8.47	30.51	35.59	20.34	5.08
COVID-19 pandemic will exacerbate economic inequalities between small and large agricultural producers	6.78	10.17	30.51	35.59	16.95
COVID-19 pandemic will decrease agricultural incomes	5.08	11.86	37.29	28.81	16.95
The consequences of COVID-19 crisis will force many producers to abandon agricultural profession	20.34	27.12	35.59	13.56	3.39
COVID-19 pandemic will cause job losses, especially in rural areas	16.95	23.73	27.12	15.25	16.95
COVID-19 pandemic will have more severe consequences in Greek farm productivity performance compared to other countries	20.34	27.12	30.51	16.95	5.08
COVID-19 economic impacts will be more deeply felt in rural areas	18.64	27.12	27.12	18.64	8.47
Unemployment rates will increase in rural areas	20.34	33.90	30.51	8.47	6.78
Social welfare will mostly decrease in rural areas	13.56	25.42	38.98	13.56	8.47

Explorative factor analysis (EFA) through principal component analysis (PCA) with varimax rotation was employed to evaluate the dimensionality of the most important factors describing experts' perceptions toward COVID-19 aftermath on agriculture and productivity. The tests that examined the quality of EFA met the common requirements. In particular, the Kaiser–Meyer–Olkin (KMO) measure was 0.880, indicating that the data employed were adequate for the PCA (77, 80). The Bartlett's test of Sphericity was highly significant (Chi-square = 427.980, *p* < 0.01) showing that the variables were correlated and suitable for structure detection. The EFA of the 11 variables provided a two-component solution and the total variance explained was 69.851%. The Cronbach's α coefficients were satisfactory since both measures were over 0.60 (81).

The results of the EFA and reliability analysis are analytically presented on Table 3. The first factor entitled as “socioeconomic impacts on rural areas” included seven variables that explained 57.170% of the total variance and had a reliability coefficient of 0.931. This factor loaded attributes related to respondents' perceptions on COVID-19 impacts on the welfare of rural regions, on farm productivity performance in isolated areas and on agricultural incomes. The second factor, “impacts on agricultural production,” incorporated four variables explaining 12.682% of the total variance with a reliability coefficient of 0.824. This factor involved attributes regarding stakeholders' concerns about the availability and accessibility to raw materials used in agriculture, the potential reduction in agro-food production and the economic inequalities between small and large farmers (Table 3).

**Table 3:** Factor analysis (PCA) and reliability analysis output on experts' perceptions toward the impact of COVID-19 crisis on agriculture.

**Eigenvalue**	**Explained variance %**	**Factors**	**Factor loading**	***m***	**S.D**.	**Cronbach's α**
		**Factor 1: Socioeconomic impacts on rural areas** COVID-19 economic impacts will be more deeply felt in rural areas	0.870	2.712	1.218	
		COVID-19 pandemic will result in job losses, especially in rural areas.	0.861	2.915	1,320	
		Unemployment rates will increase in rural areas	0.828	2.475	1.120	
6.289	57.170	Social welfare will mostly decrease in rural areas	0.813	2.780	1.152	0.931
		The consequences of COVID-19 crisis will force many producers to abandon agricultural profession	0.765	2.525	1.073	
		COVID-19 pandemic will have more severe consequences in Greek farm productivity performance compared to other EU countries	0.720	2.593	1.147	
		COVID-19 pandemic will decrease agricultural incomes	0.705	3.407	1.069	
		**Factor 2: Impacts on agricultural production** COVID-19 pandemic will cause significant shortages of raw materials used in agricultural production	0.864	2.881	1.146	
1.395	12.682	COVID-19 crisis will cause a considerable reduction in agricultural production	0.864	2.831	1.020	0.824
		COVID-19 pandemic will affect agro-food production	0.684	3.390	1.034	
		COVID-19 pandemic will exacerbate economic inequalities between small and large agricultural producers	0.630	3.458	1.069	

*m, mean; S.D., standard deviation*.

### The Impact of COVID-19 Crisis on Food Production and Food Processing Industry

Table 4 presents experts' perceptions toward COVID-19 effects on food processing business and food production. The overwhelming majority of respondents underlined the pandemic consequences on Greek economy and seemed to believe that the subsequent economic decadence will be more intense compared to the crisis on the health system (77.96%). Furthermore, respondents were convinced that the current pandemic induced crisis will mostly affect Greek economy (57.63%) but they perceived its impact on food industry as minor compared to other economic sectors (57.62%). Food experts stated that the competition between food businesses will become more intense (55.93%) with small businesses being more difficult to adjust to the turbulent economic environment. They also expressed their concerns about the viability of the small food businesses (49.15%) and the subsequent failure of many food processing businesses (55.93%). However, participants were optimistic that new opportunities could emerge in food industry since COVID-19 crisis may reveal incentives for food innovation (59.32%).

**Table 4:** Experts' perceptions toward COVID-19 impact on food processing businesses (%).

**Variables**	**Strongly disagree**	**Disagree**	**Neither disagree nor agree**	**Agree**	**Strongly agree**
COVID-19 crisis will decrease food production	16.95	25.42	32.20	20.34	5.08
COVID-19 pandemic will have a major impact on the operation of small food businesses	10.17	22.03	18.64	30.51	18.64
COVID-19 impacts will make small local food businesses less economically viable than large food businesses	11.86	15.25	23.73	23.73	25.42
COVID-19 crisis will intensify competition between businesses in food industry	6.78	11.86	25.42	33.90	22.03
COVID-19 crisis will reveal incentives for the production of innovative agricultural products	1.69	13.56	25.42	30.51	28.81
COVID-19 crisis will result in the closure of many processing businesses	10.17	20.34	13.56	35.59	20.34
Food industry will have to face a sharp decline in employment rates	13.56	16.95	25.42	18.64	25.42
COVID-19 crisis will cause significant food shortages	13.56	27.12	32.20	18.64	8.47
Greek food processing businesses lack organizational skills to cope with the growing food needs	16.95	28.81	23.73	22.03	8.47
COVID-19 crisis will have long-run impacts on food industry	6.78	22.03	28.81	33.90	8.47
COVID-19 crisis will have a minor impact on food industry compared to other industries	8.47	8.47	25.42	28.81	28.81
COVID-19 economic fallout will long outlive the health crisis	0.00	5.08	16.95	32.20	45.76
The current pandemic-induced crisis will mostly affect Greek economy compared to other countries	3.39	10.17	28.81	33.90	23.73
Food processors' interest will move toward innovative food products (e.g., functional foods)	11.86	18.64	44.07	15.25	10.17

With respect to the application of EFA in the 14-item scale, the KMO measure of sampling adequacy was 0.815, implying data suitability for the PCA (77, 80). Furthermore, the Bartlett's test of Sphericity was highly significant (Chi-square = 418.057, *p* < 0.01). Four components arose from the EFA explaining the 70.685% of the total variance. The calculated Cronbach's α reliability coefficients ranged from 0.612 to 0.892 indicating an adequate internal consistency of each factor (81).

Table 5 shows the EFA and reliability analysis output on experts' views toward the food processing businesses. The first factor labeled as “decline in the economic viability of food businesses” included five variables explaining 41.911% of the total variance and had a reliability coefficient of 0.892. This factor loaded attributes related to the competition in food industry, the severe consequences on small food businesses and the potential decrease in employment rates. The second factor labeled as “sharp economic downturn in the food industry” comprised four variables, which explained 12.247% of the total variance and had a reliability coefficient of 0.725. This factor involved attributes about the ability of food business owners to adapt themselves to the meta-COVID era and cope with individuals' food needs. The third factor included three items and was labeled as “economic recession” to describe experts' attitudes toward the impending global economic crisis and its effects on Greek economy and food production. This factor had a Cronbach's α reliability coefficient equal to 0.664 and explained 9.298% of the total variance. Finally, the fourth factor entitled as “incentives for innovation in the food sector” loaded two items regarding potential incentives for the production of innovative agricultural and food products explaining the 7.229% of the total variance and presenting a reliability coefficient of 0.612 (Table 5).

**Table 5:** Factor analysis (PCA) and reliability analysis output on experts' perceptions toward the impact of COVID-19 crisis on food production and food industry.

**Eigenvalue**	**Explained variance %**	**Factors**	**Factor loading**	***m***	**S.D**.	**Cronbach's α**
		**Factor 1: Decline in the economic viability of food businesses** COVID-19 crisis will intensify competition between businesses in food industry	0.779	3.525	1.165	
		COVID-19 impacts will make small local food businesses less economically viable than large food businesses	0.753	3.356	1.336	
5.868	41.911	COVID-19 pandemic will have a major impact on the operation of small food businesses	0.740	3.254	1.281	0.892
		COVID-19 crisis will result in the closure of many processing businesses	0.666	3.356	1.297	
		Food industry will have to face a sharp decline in employment rates	0.656	3.254	1.372	
		**Factor 2: Sharp economic downturn in the food industry** Greek food processing businesses lack organizational skills to cope with the growing food needs	0.740	2.763	1.223	
1.715	12.247	COVID-19 crisis will have major impacts on food industry compared to other industries	0.705	2.398	1.232	0.725
		COVID-19 pandemic will cause significant food shortages	0.658	2.814	1.152	
		COVID-19 crisis will have long-run impacts on food industry	0.638	3.153	1.080	
		**Factor 3: Economic recession** The current pandemic-induced crisis will mostly affect the Greek economy compared to other countries	0.798	3.644	1.063	
1.302	9.298	COVID-19 economic fallout will long outlive the health crisis	0.775	4.186	0.900	0.664
		COVID-19 crisis will decrease food production	0.509	2.712	1.130	
1.012	7.229	**Factor 4: Incentives for innovation in the food industry** COVID-19 crisis will reveal incentives for the production of innovative agricultural products	0.894	3.712	1.084	0.612
		Food processors' interest will move toward innovative food products (e.g., functional foods)	0.663	2.932	1.112	

*m, mean; S.D., standard deviation*.

### The Impact of COVID-19 Crisis on Food Distribution Networks

Respondents' perceptions toward the consequences of the pandemic on food distribution channels are described on Table 6. The vast majority of agro-food experts were convinced that COVID-19 crisis will substantially result in higher food production costs, leading in turn to higher retail prices of food products (52.54%). Furthermore, ~56% of participants believed that overloaded transport networks may lead to market shortages of perishable agro-food products (55.93%), whereas nearly 53% agreed that foods may be delivered to consumers after the scheduled time. A significant proportion of respondents expressed their concerns about the export and import volume of agricultural/livestock products, considering that there will be a noticeable decrease of both in the meta-COVID era (Table 6).

**Table 6:** Experts' perceptions toward COVID-19 impact on food distribution networks (%).

**Variables**	**Strongly disagree**	**Disagree**	**Neither disagree nor agree**	**Agree**	**Strongly agree**
COVID-19 pandemic will lead to significant shortages of agro-food products	15.25	20.34	37.29	20.34	6.78
I believe that the retail prices of agro-food products will considerably increase in the near future	6.78	16.95	23.73	37.29	15.25
I believe that there will be significant food shortages in regions far from urban areas	13.56	28.81	25.42	22.03	10.17
Food production costs will dramatically increase	5.08	16.95	25.42	38.98	13.56
Overloaded transport networks may lead to market shortages of perishable agro-food products (e.g., grocery)	6.78	18.64	18.64	38.98	16.95
The pressure on distribution networks may reduce food quality	15.25	16.95	32.20	23.73	11.86
COVID-19 pandemic will reduce the ability of the supply chain systems to deliver products to consumers in a timely manner	8.47	15.25	23.73	42.37	10.17
COVID-19 pandemic will lead to a reduction in the exports of Greek agricultural-livestock products	10.17	13.56	25.42	30.51	20.34
COVID-19 pandemic will lead to a reduction in imports of agricultural/livestock products	6.78	11.86	23.73	47.46	10.17

EFA revealed the most important components that depict experts' perceptions toward the pandemic effects on food distribution. Two factors were extracted explaining 71.066% of the total variance, whereas the KMO measure with a value of 0.861 and the Bartlett's test of Sphericity (Chi-square = 306.198, *p* < 0.01) met the common requirements and justified the implementation of EFA through PCA with varimax rotation (77, 81). The Cronbach's α coefficients were satisfactory with values over 0.60 (81).

The first factor accounted for 55.908% of the total variance and was characterized by four of the nine variables that constructed the 5-point scale. This first factor reflected experts' views on the consequences of COVID-19 crisis on distribution systems efficiency and was labeled as “distribution channels fallout” (“the pressure on distribution networks may reduce food quality,” “COVID-19 crisis will reduce the supply chain's ability to deliver products to consumers in a timely manner,” “Overloaded transport networks may lead to market shortages of perishable agro-food products (e.g., grocery),” “Food production costs will dramatically increase”). Reliability analysis provided the Cronbach's α calculation equal to 0.890. The second factor accounted for 15.158% of the total variance and was characterized by five of the nine variables (“COVID-19 pandemic will lead to a reduction in imports of agricultural/livestock products,” “I believe that the prices of agricultural products will considerably increase in the near future,” “I believe that there will be significant food shortages in regions far from urban areas,” “COVID-19 pandemic will lead to significant shortages of agricultural products,” “COVID-19 pandemic will lead to a reduction in the exports of Greek agricultural/livestock products”). This factor was labeled as “food supply disruption” and the Cronbach's α coefficient had a value of 0.842 (Table 7).

**Table 7:** Factor analysis (PCA) and reliability analysis output on experts' perceptions toward the impact of COVID-19 crisis on food distribution networks.

**Eigenvalue**	**Explained variance %**	**Factors**	**Factor loading**	***m***	**S.D**.	**Cronbach's α**
		**Factor 1: Distribution channels fallout** The pressure on distribution networks may reduce food quality	0.873	3.000	1.232	
5.032	55.908	COVID-19 crisis will reduce the supply chain's ability to deliver products to consumers in a timely manner	0.849	3.305	1.119	0.890
		Overloaded transport networks may lead to market shortages of perishable agro-food products (e.g., grocery)	0.843	3.407	1.176	
		Food production costs will dramatically increase	0.746	3.390	1.083	
		**Factor 2: Food supply disruption** COVID-19 pandemic will lead to a reduction in imports of agricultural and livestock products (e.g., milk)	0.86	3.424	1.053	
		I believe that the retail prices of agro-food products will considerably increase in the near future	0.702	3.373	1.143	
1.364	15.158	I believe that there will be significant food shortages in regions far from urban areas	0.687	2.864	1.210	0.842
		COVID-19 pandemic will lead to significant shortages of agro-food products	0.668	2.831	1.132	
		COVID-19 pandemic will bring a reduction in the exports of Greek agricultural—livestock products	0.645	3.373	1.244	

*m, mean; S.D., standard deviation*.

### The Impact of COVID-19 Crisis on Consumers' Food Preferences

Experts' perceptions toward the impact of COVID-19 crisis on consumers' food habits are reported on Table 8. Most of the participants believed that the pandemic will bring changes to consumers' food preferences and consumption patterns with noticeable increases in the demand for long shelf-life products (76.27%), packaged foods (84.74%), and locally-produced foods (76.27%). Furthermore, consumers may become more oriented toward ready-to-eat meals (44.07%), innovative (38.98%), and sustainable food products (49.15%). Although a significant proportion of participants were convinced that the pandemic crisis may turn consumers' interest to healthier eating choices and enhance the prevalence of the Mediterranean diet, a significant proportion of respondents considered that it is very possible that consumers' interest for cheaper and unhealthy food options will increase.

**Table 8:** Experts' perceptions toward COVID-19 impact on consumers' food habits and preferences (%).

**Variables**	**Extremely unlikely**	**Unlikely**	**Neutral**	**Likely**	**Extremely likely**
The demand for foods with long shelf-life will increase	1.69	5.08	16.95	47.46	28.81
Consumers will become more demanding with the measures taken by stakeholders to protect public health	1.69	5.08	8.47	45.76	38.98
The pandemic will increase food e-commerce	0.00	1.69	10.17	27.12	61.02
COVID-19 crisis will increase the consumption of packaged agri-food products	1.69	3.39	8.47	33.90	52.54
COVID-19 crisis will increase the demand for ready-to-eat meals	8.47	25.42	22.03	30.51	13.56
Consumers will become more skeptical about fresh foods	3.39	10.17	22.03	25.42	38.98
COVID-19 crisis may turn consumers to unhealthy food choices	11.86	27.12	16.95	33.90	10.17
Consumers will turn to locally-produced foods that are easier to obtain	0.00	6.78	16.95	33.90	42.37
COVID-19 crisis will turn consumers to adopt Mediterranean diet	1.69	10.17	35.59	25.42	27.12
COVID-19 crisis will increase consumers' attention to health issues	0.00	0.00	11.86	37.29	50.85
COVID-19 crisis will turn consumers to cheaper food choices	5.08	18.64	38.98	23.73	13.56
COVID-19 crisis may increase demand for innovative food products (e.g., super-foods, functional foods, etc.)	10.17	16.95	33.90	25.42	13.56
COVID-19 crisis may increase consumer demand for sustainable food products (e.g., organics)	6.78	8.47	35.59	33.90	15.25

EFA revealed three factors that explain ~63.50% of the total variance, whereas the KMO test for sampling adequacy had a value of 0.778 that was above the acceptable level of 0.60. The Bartlett's test of Sphericity (Chi-square = 321.540, *p* < 0.01) also justified the implementation of EFA through PCA with varimax rotation (77, 81). The Cronbach's α coefficients were satisfactory with values ranging from 0.610 to 0.857 (81).

The first factor accounted for 35.944% of the total variance and included eight of the 13 items describing experts' viewpoints on consumers' awareness for food safety and consciousness toward health issues. This first factor was characterized as “consumers' increasing interest in health protection” and had a reliability coefficient of 0.857. The second factor accounted for 18.623% of the total variance and was characterized by three of the 13 variables presenting participants perceptions toward consumers' food choices and preferences in the meta-COVID era. Food experts seemed to believe that consumers were more likely to shift to convenience and cheaper meals and hence the second factor was entitled as “adoption of unhealthy eating habits” and had a reliability coefficient equal to 0.679. Finally, the third factor was found to explain 8.922% of the total variance and was labeled as “consumers' demand for innovative and sustainable foods” according to the content of the two variables included. Reliability analysis provided a Cronbach's α measure equal to 0.610 (Table 9).

**Table 9:** Factor analysis (PCA) and reliability analysis output on experts' perceptions toward the impact of COVID-19 crisis on consumers' food habits.

**Eigenvalue**	**Explained variance %**	**Factor**	**Factor loading**	***m***	**S.D**.	**Cronbach's α**
		**Factor 1: Consumers' increasing interest in health protection** Consumers will become more demanding with the measures taken by stakeholders to protect public health	0.834	4.153	0.906	
		COVID-19 crisis will increase consumers' attention to health issues	0.784	4.390	0.695	
		Consumers will turn to locally-produced foods that are easier to obtain	0.675	4.120	0.930	
4.673	35.944	COVID-19 crisis will increase the consumption of packaged agri-food products	0.661	4.322	0.899	0.857
		COVID-19 crisis will turn consumers to adopt Mediterranean diet	0.654	3.661	1.044	
		The demand for foods with long shelf-life will increase	0.650	3.966	0.909	
		Consumers will become more skeptical about fresh foods	0.604	3.864	1.152	
		The pandemic will increase food e-commerce	0.594	4.475	0.751	
		**Factor 2: Adoption of unhealthy eating habits** The COVID-19 crisis will turn consumers to cheaper food choices	0.748	3.220	1.068	
2.421	18.623	COVID-19 crisis will increase the demand for ready-to-eat meals	0.740	3.153	1.201	0.679
		COVID-19 crisis may turn consumers to unhealthy food choices	0.726	3.034	1.231	
1.160	8.922	**Factor 3: Consumers' demand for innovative and sustainable foods** COVID-19 crisis may increase demand for innovative food products (e.g., super-foods, functional foods, etc.)	0.888	3.153	1.172	0.61
		COVID-19 crisis may increase consumer demand for sustainable food products (e.g., organics)	0.632	3.424	1.070	

*m, mean; S.D., standard deviation*.

**Figure 1:**
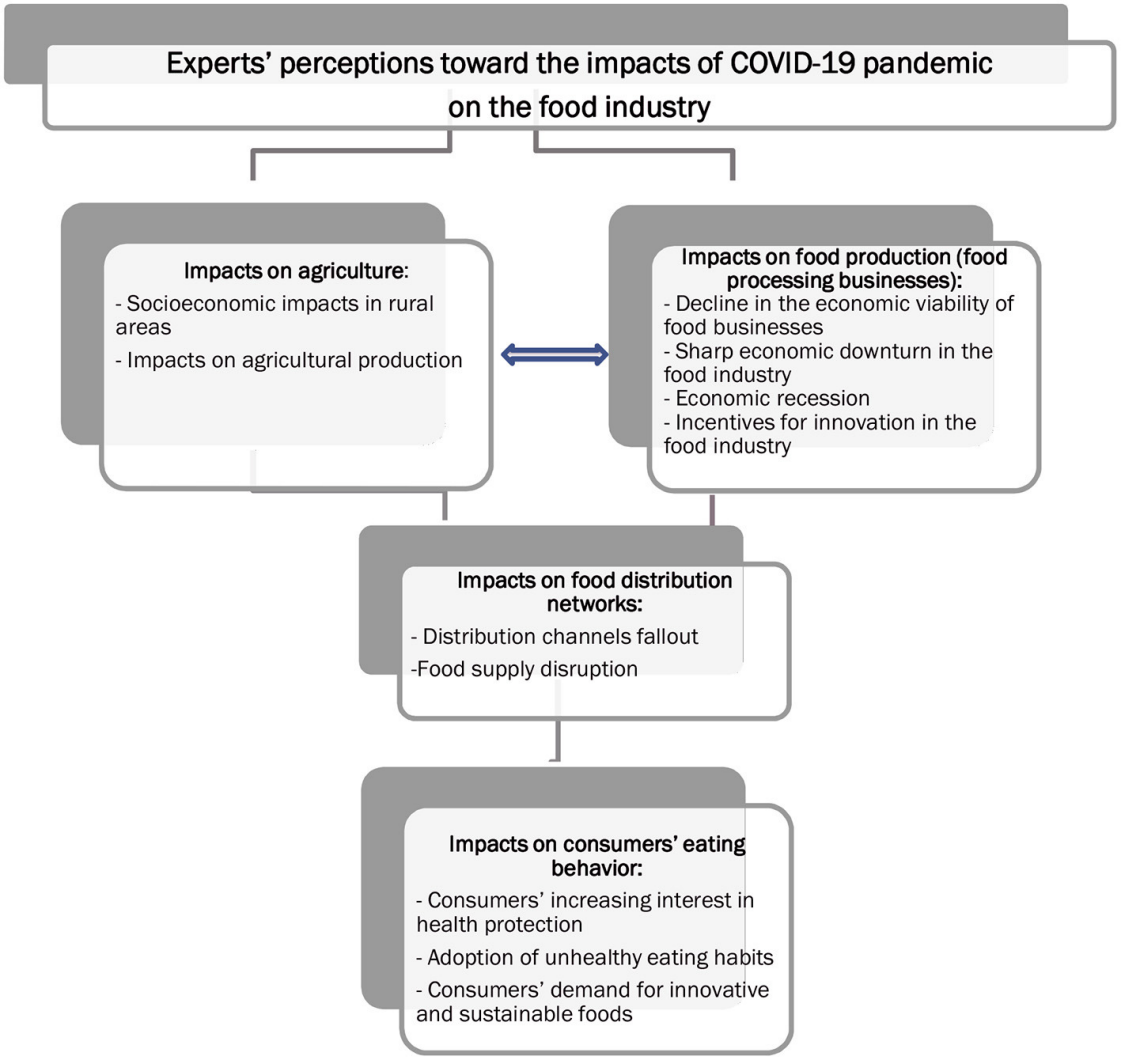
Experts' perceptions toward the impacts of COVID-19 pandemic on the various facets of the food industry.

Figure 1 summarizes the results derived from the EFA application and provides a thorough presentation of the experts' perceptions toward the impacts of COVID-19 pandemic on the various facets of the food industry, from the production stage to the final purchase and consumption of the agro-food products.

### Cluster Analysis Results

Cluster analysis was conducted on two stages to classify experts' segments on the basis of their attitudes toward the impact of COVID-19 on agriculture, food processing industry, food distribution networks, and consumers' food habits and preferences. Cluster analysis resulted to the identification of two expert segments. *T*-tests for the equality of means indicated statistically significant differences between the two clusters in terms of perceptions toward agro-food production and food business viability, food distribution problems, and potential changes in food consumption patterns. The clusters were labeled according to the factors that were considered to be of high importance for each of them (Table 10). To provide a complete profile of food expert segments, cross-tabulation and Pearson's χ^2^ statistics were also estimated to define differences in business characteristics between clusters (Table 11).

**Table 10:** Cluster analysis results for experts' attitudes toward COVID-19 consequences for food industry.

**Factors**	**Total sample**		**Cluster 1 (33.9%)**		**Cluster 2 (66.1%)**		***t*** **-test for equality of means**	
	**Mean**	**S.D**.	**Mean**	**S.D**.	**Mean**	**S.D**.	***t*** **-test**	***P*** **-value**
Socioeconomic impacts on rural areas	2.772	0.972	1.836	0.596	3.253	0.752	−7.321	0.000
Impacts on agricultural production	3.140	0.871	2.325	0.730	3.558	0.603	−6.915	0.000
Decline in the economic viability of small food enterprises	3.349	1.080	2.290	0.907	3.892	0.690	−7.576	0.000
Sharp economic downturn in the food industry	2.780	0.869	2.062	0.688	3.147	0.711	−5.608	0.000
Economic recession	3.514	0.801	2.916	0.844	3.821	0.582	−4.831	0.000
Incentives for innovation in the food industry	3.322	0.932	2.800	0.849	3.590	0.865	−3.340	0.001
Distribution channels fallout	3.275	1.001	2.500	1.020	3.673	0.728	−5.098	0.000
Food supply disruption	3.173	0.907	2.310	0.724	3.616	0.663	−7.140	0.000
Consumers' increasing interest in health protection	4.119	0.651	3.775	0.809	4.300	0.474	−3.116	0.003
Adoption of unhealthy eating habits	3.136	0.912	2.750	0.930	3.334	0.848	−2.421	0.019
Consumers' demand for innovative and sustainable foods	3.288	0.948	2.975	0.938	3.449	0.923	−1.855	0.069

*S.D., standard deviation*.

**Table 11:** Cluster profile (*N* = 59).

	**Variables**	Cluster 1^*^(33.9%)	Cluster 2^*^(66.1%)	**Pearson chi-square**	***P*** **-value**
Occupation	Farmers	20.0	30.8		
	Food processing enterprises	35.0	33.3	1.504	0.681
	Food distributors—sellers	25.0	25.6		
	Consultants—policy makers	20.0	10.3		
	Micro enterprises (up to 10 employees)	26.3	51.3		
Enterprise size	Small-sized enterprises (11–49 employees)	42.1	20.5	4.301	0.231
	Medium sized enterprises (50–250 employees)	21.1	15.4		
	Large enterprises (over 250 employees)	10.5	12.8		
COVID-19 pandemic has affected the	No	20.0	0	8.367	0.004
business/organization I work for	Yes	80.0	100.0		
To what extent do you think the	Not at all	20.0	0		
activity of the business/organization in	Slightly	15.0	0		
which you are employed has been	Moderately	35.0	25.6	18.577	0.001
affected by COVID-19 pandemic?	Very much	20.0	56.5		
	Extremely	10.0	17.9		
How the economic performance of	It will deteriorate	55.0	89.7		
the business/organization, in which	It will not be affected	25.0	2.6	10.279	0.006
you work, will be affected in the near future?	It will improve	20.0	7.7		

* *Percentage*.

Cluster 1 was labeled as “skeptical to COVID-19 impacts” and included 33.9% of respondents. This segment had the lowest mean scores in “socioeconomic impacts on rural areas” (1.836 vs. 3.252, *t*-test = −7.321, *p* < 0.01), “decline in the economic viability of small food enterprises” (2.290 vs. 3.892, *t*-test = −7.576, *p* < 0.01) and “sharp economic downturn in the food industry” (2.062 vs. 3.147, *t*-test = −5.608, *p* < 0.01) factors compared to Cluster 2. Food experts that represented Cluster 1 had reservations on the pandemic effects on agricultural and food production and were skeptical about potential changes in consumers' food patterns as a direct impact of the pandemic on food preferences and choices (Table 10). The first cluster grouped food experts who believed that the pandemic had affected the activity of the enterprise/service in which they were employed (80.0%), although they were convinced that its economic performance will either remain stable or even improve in the near future (45.0%) (Table 11).

Cluster 2 represented “alarmed food experts about COVID-19 impacts” and included 66.1% of participants. The members of this cluster were convinced that COVID-19 aftermath will be severe for both the agro-food sector and the economy since they scored highly on the “impacts on agricultural production” (3.560 vs. 2.325, *t*-test = −6.915, *p* < 0.01), “economic recession” (3.821 vs. 2.904, *t*-test = −4.831, *p* < 0.01) and “distribution channels fallout” (3.673 vs. 2.500, *t*-test = −5.098, *p* < 0.01). Furthermore, cluster 2 participants agreed that there is an increasing risk for consumers' to adopt less healthy eating habits (3.334 vs. 2.750, *t*-test = −2.421, *p* < 0.05), although they noted the emerging opportunities for food innovation in the food sector and the rising demand for innovative and sustainable food choices. All the food experts in this cluster declared that the pandemic has clearly affected the activity of their enterprise, whereas 89.7% agreed that its economic performance will deteriorate in the short-run (Table 11). Finally, discriminant analysis confirmed the classification accomplished through cluster analysis, showing that the exactness of classification was 98.3%.

## Discussion

The present study sought to explore the aftermath of the COVID-19 pandemic on the food sector. For this reason an explorative research was designed to select information from a sample of food experts regarding the impact of COVID-19 crisis on all the dimensions of the food industry, including agro-food production, agro-food products distribution and delivery, and consumers' food purchase patterns. Considering the catalytic role of the new pandemic in the agro-food environment, food experts were deemed the most appropriate to highlight the new reality in the food chain and assess evidence whether it will ever revert to pre-COVID-19 “norms.”

### COVID-19 Pandemic and Its Impact on Agriculture and Agricultural Businesses

Our findings showed that the pandemic is expected to have major impacts on the agricultural sector. A great proportion of experts agreed that the COVID-19 breakout may exacerbate economic inequalities between small and large producers and strike farmers' incomes to such an extent that may cause job losses and increase unemployment rates, especially in rural areas. Although experts seemed to be rather cautious to predict a subsequent reduction in agricultural production, they were convinced that the meta-COVID-19 era will signify crucial modifications in the food sector. Besides, the majority (66.1%) were skeptical to the pandemic repercussions affirming its socioeconomic impacts and the welfare decrease in rural areas. Other studies have also indicated the pandemic consequences in agricultural production underlying their reflection to the root of the food system. For instance in India, wheat and pulse harvesting were hindered due to migrant workers' shortage, whereas in Ethiopia, farmers had to deal with income loss due to overstocked produce and input shortage (82–84).

It is a common practice in many countriess that seasonal farmworkers are usually employed for planting, sorting,s harvesting, processing, and transporting cultivated crops (13). During the pandemic, workers' absenteeism because of COVID-19 infection or transportation restrictions imposed by repeated lockdowns made farm productivity more fragile, especially in labor intensives sectors, such as planting and harvesting, horticulture, and livestock productions (13, 14, 85). Reduced capacity in agricultural supply resulted in volatility and increases in agricultural prices, putting extra burdens on retail price for agricultural commodities and tightening consumer purchase power. For some agricultural products, such as meat, recent evidence showed that although wholesale and retail meat prices were surged to peak, livestock prices were decreasing (86, 87). This boost in the farm-to-wholesale marketing margin (87) could have major impacts on the welfare of both producers and consumers.

Due to the nature of the agricultural activity, there is a specific timetable to follow for maintaining stability in the production process. Since the start of COVID-19 outbreak, many farmers had no other choice left but destroy their products because of the restrictive measures. According to the Dairy Farmers of America, farmers were forced to dump ~3.7 million gallons of milk every day and chicken processors faced serious problems with staff absenteeism and had to euthanize chickens because of the reduced capacity in plants (88, 89). Under such conditions, farmers have to come to terms with income uncertainty, whereas the reduction of consumers' purchasing power may further push farmers' incomes downwards in the long term (90, 91). This shrinkage of farmers's incomes could increase the risks for lower quality of the production and undermines public health protection since some farmers might move toward irregular practices to decrease expenses associated with crop protection (e.g., fertilizer and pesticide usage) and livestock health (e.g., disease control in farm animals).

On the other hand, COVID-19 crisis benefited small producers over large agricultural companies from consumers' increasing interest on online purchases of agricultural products (92). At the same time, the engagement of small producers in online trading platforms helped satisfy the urge of consumers for variety seeking and their need for locally produced agricultural commodities.

### COVID-19 Pandemic and Its Impact on Food Processing Businesses

Food experts in this study also noted the severe impacts of COVID-19 on food production, underlining the increasing risks for the economic viability of the small food businesses, the escalated competition in the food industry, and after all the economic failure and closure of a significant number of food enterprises in the long-run. The burden on distribution networks and the increase in production costs will affect food retail prices that are expected to climb up in higher levels compared to the pre-pandemic levels. This increase in food retail prices may cause changes in consumer purchase behavior, turning potential buyers to cheaper food options, even of lower quality. Food experts also questioned the food industry capacity to meet consumers' needs for perishable food products and highlighted the potential of food quality reduction due to the burden on the distribution networks and market shortages. Inadequate food availability, especially in perishable commodities (such as fruits, vegetables, dairy products), in conjunction with the subsequent increases in food prices might oblige a significant proportion of consumers to substitute fresh products with ultra- processed foods and adopt less healthy eating patterns.

Within the food sector, micro-, small-, and medium-sized enterprises (MSMEs) have a crucial role in food systems by providing “almost half of total calories worldwide” (93, 94). Since, most of the Greek food enterprises are SMEs and employ ~85% of total workforce (79), any disruption in their operation will have a critical impact on Greek economy. SMEs have been reported as the major victims of the COVID-19 pandemic because in comparison with the large enterprises, they have fallen short of financial and managerial resources and have also been unprepared to handle and overcome uncertainties created by extreme events or natural hazards (30). In addition, food processing enterprises seemed to comprise “hot spots” for the pandemic expansion since social distancing measures were hard to implement inside the food plants during the long shifts, or even outside the food plants where employees traveled on the same bus or train or used car-sharing systems to reduce transportation costs (13). Recent evidence indicated that SMEs usually organize short-term planning, have less strict protocols to react to unexpected situations and mostly pay attention on nothing more than their economic survival (95, 96). Sullivan-Taylor and Branicki (97) explained that the limited managerial resources and financial constraints of the SMEs may hinder their ability to adapt to risk management strategies, and hence decrease their resilience to extreme situations, such as the current pandemic.

Food experts also brought up the emerging opportunities for innovation in the agro-food sector. COVID-19 crisis generated challenges for the market of innovative functional foods designed to boost consumers' immune system through a combination of target bioactive compounds, such as vitamins and antioxidants in order to avoid infections and improve overall health (98). Recent evidence showed that research and innovative practices have also recovered bioactive compounds from processed byproducts to replace synthetic additives with natural health-stimulating ingredients (99). Following consumers' health priorities, food companies will have to adjust to the new trends for the commercialization and promotion of food products with health benefits (21) and also meet the increasing demand for nutraceuticals, healthier meal types, and home-prepared foods (13). This shift in consumer purchase behavior signifies a major change in prevalent health standard and a “move from a curative to a preventive model” (100).

### COVID-19 Pandemic and Its Impact on Food Consumption

This study also showed that COVID-19 outbreak altered consumers' food habits and preferences. The demand for long shelf-life and packaged foods was expected to increase since consumers became more skeptical about fresh foods. Our findings further support previous research noting that during lockdown periods individuals shopped less frequently and increased the consumption of longer shelf-life foods, such as dried or canned foods, pasta, milk and frozen foods, due to convenience, and daily cooking at home (13). On the other hand, there was a noticeable decrease in fresh food consumption because consumers hesitated to visit food and grocery stores and perceived packaged foods as more hygienic (32, 101). The pandemic has also spread food e-commerce since consumers were forced to turn to online food delivery systems for avoiding personal contacts in food stores (102). Furthermore, feelings of uncertainty about the availability of specific food choices in food stores and lack of confidence for shopping skills also motivated consumers to use online food services (13, 103).

COVID-19 induced psychological changes might also influence food purchase behaviors. Food experts in our study highlighted the subsequent modifications in food choices and the adoption of unhealthy eating habits. When individuals were exposed to extensive communication about the pandemic health risks, stress, and anxiety symptoms were triggered. In attempt to regulate anxiety, many consumers increased the consumption of processed “comfort foods,” such as chocolate and chips, whereas others tried to make themselves think positive by eating or drinking when under stress (32, 101, 104–106). Furthermore, the unhealthy eating patterns during the quarantine period increased health risks, especially for overweight and obese individuals. Several studies reported that significant proportions of the population gained weight because of the higher consumption of more processed and energy dense foods, snacking frequency increase, and physical activity decrease (32, 106, 107). To protect public health and maintain dietary balance during the pandemic, nutrition, and health information programs should provide incentives for cooking at home and enhance the consumption of meals prepared with fresh and less processed ingredients. The food market constitutes an important element of the economy whose viability largely depends on consumer viewpoints and trust in the food system. How consumers receive food-purchasing decisions in times of uncertainty has a critical impact for both the agro-food sector and the whole economy.

### Study Limitations

Although the present study sought to investigate disruptions in all the aspects of the food system, there are some limitations that should be reported. First, the data set selected for this study included self-reported information, which might be affected by reporting bias (108). Stakeholders who were more affected by COVID-19 consequences, they may be more likely to express more pessimistic views of the food industry's future. In addition, the cross sectional design does not allow generating relationships in the long run (109). Data selection started after the first lockdown, when most food businesses had to adapt to the new reality and overcome obstacles in the food chain. Longitudinal follow-ups may provide a thorough picture of the COVID-19 aftermath and illustrate the emerging opportunities for the food industry in the meta-pandemic era. Furthermore, food experts were recruited during the period of the first quarantine when extra pressure was put on food managers to ensure food availability and safety standards. It is possible that potential respondents refused to participate in this survey, which might result in data loss and significant differences between those who responded and those who did not. Although our sample size could be considered as small, the response rate is quite satisfactory and significantly higher to the average response rate of 11% for the online surveys (48). However, we believe that a replication of the present study in the near future could attract more participants and enhance the validity of our findings.

### Policy/Practical Implications

This pandemic has offered a unique opportunity to learn more about the fragility of the food environment and increase readiness to cope with future disruptions. COVID-19 crisis will likely continue to interrupt the operation of the food industry in the near future and food businesses will have to consider the possibility of new food system disruptions in their strategic plans, investment, and managerial efficacy. For instance, dairy farmers in China transferred their milk production to processing services in order to process it to milk powder for storage during the pandemic (110). Training programs and seminars directed to the food industry could help food managers and employees adapt to potential food chain disruptions and develop strategic plans for their most appropriate allocation of their resources and also invest in processing facilities, which may increase the storage facility, especially of perishable foods. Agricultural cooperatives could also train their members to deal with price fluctuations and instability in agricultural production. Beyond its negative consequences in the food system, the pandemic has created opportunities for innovation and the development of communication technologies that can be implemented to improve agro-food production and food supply systems and also minimize food loss and waste. COVID-19 outbreak made it clear that food systems have to adjust to the new turbulent environment and increase food security. Effective responses to the pandemic should first ensure that global food systems remain open and operational, so that food can be delivered where it is needed, even in deprived regions. Global cooperation is necessary to speed up border procedures and ensure the stability of the global supply chains. Food policy should also be oriented toward making agricultural and food systems more sustainable and resilient in order to help societies transition toward a climate-neutral economy.

## Data Availability Statement

The datasets presented in this article are not readily available because the present study analyzed data selected from a sample of food experts. Participants engaged in the survey voluntarily and confidentiality of their responses was stated in the invitation letter. In particular, respondents were assured that data would remain confidential and would not be shared. Therefore, data (data set is in Greek) could be given only in exceptional circumstances (and after reasonable request). Requests to access the datasets should be directed to ER, elenra@agro.duth.gr.

## Ethics Statement

Ethical review and approval was not required for the study on human participants in accordance with the institutional requirements. The patients/participants provided their written informed consent to participate in the study.

## Author Contributions

ER designed the formal questionnaire used for data selection, conducted the statistical analysis, and wrote the manuscript draft. All authors contributed to the study concept. All authors edited and approved the submitted manuscript.

## Conflict of Interest

The authors declare that the research was conducted in the absence of any commercial or financial relationships that could be construed as a potential conflict of interest.

## Publisher's Note

All claims expressed in this article are solely those of the authors and do not necessarily represent those of their affiliated organizations, or those of the publisher, the editors and the reviewers. Any product that may be evaluated in this article, or claim that may be made by its manufacturer, is not guaranteed or endorsed by the publisher.
